# Deciphering the Role of Fluoroethylene Carbonate towards Highly Reversible Sodium Metal Anodes

**DOI:** 10.34133/2022/9754612

**Published:** 2022-01-27

**Authors:** Xueying Zheng, Suting Weng, Wei Luo, Bo Chen, Xiao Zhang, Zhenyi Gu, Haotian Wang, Xiaolu Ye, Xuyang Liu, Liqiang Huang, Xinglong Wu, Xuefeng Wang, Yunhui Huang

**Affiliations:** ^1^Institute of New Energy for Vehicles, Shanghai Key Laboratory of Development & Application for Metallic Functional Materials, School of Materials Science and Engineering, Tongji University, Shanghai 201804, China; ^2^Institute of Physics, Chinese Academy of Sciences; School of Physical Sciences, University of Chinese Academy of Sciences, Beijing 100190, China; ^3^Key Laboratory of Advanced Civil Engineering Materials (Tongji University), Ministry of Education, Shanghai 201804, China; ^4^Key Laboratory for UV Light-Emitting Materials and Technology, Northeast Normal University, Ministry of Education, Changchun, Jilin 130024, China; ^5^Tianmu Lake Institute of Advanced Energy Storage Technologies Co., Ltd., Liyang, Jiangsu 213300, China; ^6^State Key Laboratory of Materials Processing and Die & Mould Technology, School of Materials Science and Engineering, Huazhong University of Science and Technology, Wuhan, Hubei 430074, China

## Abstract

Sodium metal anodes (SMAs) suffer from extremely low reversibility (<20%) in carbonate-based electrolytes—this piece of knowledge gained from previous studies has ruled out the application of carbonate solvents for sodium metal batteries. Here, we overturn this conclusion by incorporating fluoroethylene carbonate (FEC) as cosolvent that renders a Na plating/stripping efficiency of >95% with conventional NaPF_6_ salt at a regular concentration (1.0 M). The peculiar role of FEC is firstly unraveled via its involvement into the solvation structure, where a threshold FEC concentration with a coordination number>1.2 is needed in guaranteeing high Na reversibility over the long-term. Specifically, by incorporating an average number of 1.2 FEC molecules into the primary Na^+^ solvation sheath, lowest unoccupied molecular orbital (LUMO) levels of such Na^+^-FEC solvates undergo further decrease, with spin electrons residing either on the O=CO(O) moiety of FEC or sharing between Na^+^ and its C=O bond, which ensures a prior FEC decomposition in passivating the Na surface against other carbonate molecules. Further, by adopting cryogenic transmission electron microscopy (cryo-TEM), we found that the Na filaments grow into substantially larger diameter from ~400 nm to >1 *μ*m with addition of FEC upon the threshold value. A highly crystalline and much thinner (~40 nm) solid-electrolyte interphase (SEI) is consequently observed to uniformly wrap the Na surface, in contrast to the severely corroded Na as retrieved from the blank electrolyte. The potence of FEC is further demonstrated in a series of “corrosive solvents” such as ethyl acetate (EA), trimethyl phosphate (TMP), and acetonitrile (AN), enabling highly reversible SMAs in the otherwise unusable solvent systems.

## 1. Introduction

Metallic sodium manifests an ultimate anode for sodium-based batteries due to its highest theoretical specific capacity (1165 mAh g^−1^), lowest redox potential (−2.71 V versus standard hydrogen electrode), and a wide geographical availability [1, 2]. Unfortunately, using the conventional electrolytes prevailing for sodium-ion batteries (SIBs), typically the mixed carbonate solvents with 1 M NaPF6 or NaClO4, SMAs are known to experience dendritic and porous deposition, entailing massively rising Na exposure area that exacerbates the parasitic reactions between Na and the electrolyte [3, 4]. Consequently, Na sources are intensively lost during this process to form thick and nonuniform byproducts covering on each individual Na deposit. During Na stripping, the tortuous structure of Na deposits, along with the largely resistive SEI formed in carbonate electrolytes, makes it prone to lose the conductive paths from the dendrite tip to the base [5, 6]. Such detached Na then turns to dead Na, constituting another major cause for the low CE (<20%) observed in carbonate electrolytes [7]. This vicious cycle pertains with subsequent cycling, gradually blocks the interfacial Na^+^ transfer, depletes the electrolyte, and triggers an early cell failure accordingly.

Finding the way out, Cui et al. found that the ether solvents (eg., diglyme, triglyme, or tetraglyme) are particularly compatible with SMAs, enabling a Na plating/stripping CE of >99% with 1 M NaPF_6_ [8]. Such CE improvement was majorly brought down to a Na-O and NaF-rich SEI, which efficiently guards Na against subsequent side-reactions and the volume changes. However, for that ether solvents are plagued by the high-volatility, high-flammability, and a low oxidative stability, their practical applications in sodium metal batteries (SMBs) are still deterred especially when pairing with a higher-voltage cathode [9, 10]. Thus, it remains particularly appealing if we can enable reversible Na plating/stripping with the conventional electrolyte recipes used in SIBs.

Since both Na plating and stripping take place through the SEI formed on Na surface, formulating a carbonate-based electrolyte that forms a uniform and passivating SEI on SMA would therefore constitute a most effective avenue in achieving desirable efficiencies [11, 12]. The magic role of FEC additive has long been confirmed in both Li-ion and Na-ion battery systems for its film-formation capability on the anode side [13–16]. In recent years, FEC has also shown its efficacy for Li metal batteries, where steadily enhanced Li CEs are accompanied with stabilized interfacial resistance [17, 18]. The underlying causes, however, are still under debate which are often vaguely attributed to the LiF-rich SEI generated with prior defluorination of FEC. Being an analogue to Li, the SEI formed on SMA should also benefit from the introduction of FEC. Nevertheless, instead of directly borrowing the experiences from Li-ion, Na-ion, or Li metal battery systems, SMA definitely requires its specific FEC dosage for its much higher reactivity than the other anode materials, such as hard carbon.

**Figure 1:**
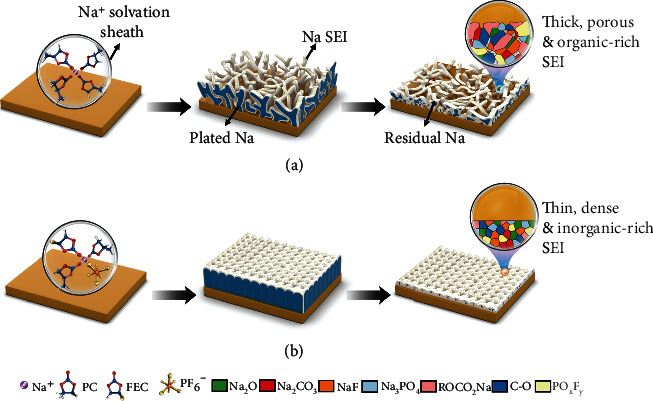
Schematics showing the Na plating/stripping morphology with enlarged SEI structures. (a) Using the conventional carbonate-based electrolyte, Na plates dendritically with high tortuosity and a thick, organic-rich SEI, making Na deposits readily lose their electrical connection during the stripping process. (b) With FEC coordinated above the threshold value, Na plates into much larger size with minimum tortuosity, the thin and inorganic-rich SEI further promotes a uniform Na stripping, significantly boosting the Na plating/stripping efficiency.

While this field still lacks a systematic investigation that unravels the role of FEC for SMAs in correlation with its optimum concentrations. In this work, we fill this knowledge gap by exploring the cosolvation of FEC in the conventional propylene carbonate- (PC-) based electrolyte and its consequences on the performances of SMBs. Through balancing the Na CE, interfacial resistance, full cell performance, and physical properties of the electrolytes, a threshold FEC concentration is established in connection with its influence on Na^+^ solvation structure. Specifically, we show the coordination number of FEC should exceed ~1.2 to guarantee an adequate FEC decomposition to passivate the Na surface. As such, the Na deposits evolve into much larger filaments with a denser structure, which not only mitigate the Na exposure area to the electrolyte but also render a tight structural connection between the Na pieces. By combining Cryo-TEM with indepth XPS measurements, a highly crystalline SEI is distinguished on SMA possessing rich inorganics such as NaF, Na_2_O, and Na_2_CO_3_, in contrast to the thick and largely amorphous SEI formed in the FEC-free counterpart (Figure 1). Moreover, the protocol established herein also applies for EA, TMP, and AN-based electrolytes, denoting the role of FEC in promoting highly-reversible SMAs in a wide variety of the electrolyte systems.

## 2. Results and Discussion

### 2.1. FEC Concentration-Dependent Na Metal Performances

**Figure 2:**
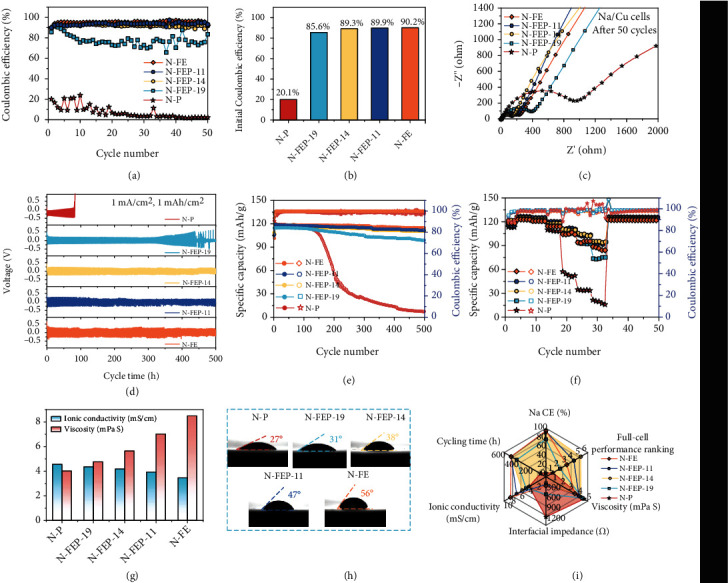
Evaluation comparing the overall battery performances with gradient FEC concentrations. (a) CE for Na plating/stripping using the corresponding electrolyte in Na/Cu cell configuration. The cycling was conducted at 0.5 mA/cm^2^ and 1.0 mAh/cm^2^. (b) Bar chart illustrating the ICE for Na plating/stripping using the corresponding electrolyte. (c) Nyquist plots of the Na/Cu cells after 50 cycles. (d) Long-term cycling performance of Na metal in Na/Na symmetrical cell configuration at 1.0 mA/cm^2^ and 1.0 mAh/cm^2^. (e) Cycling performance of the Na/NVPOF full cells for 500 cycles at 1 C. (f) Rate behavior of the Na/NVPOF full cells at increasing rates from 1 C to 20 C. (g) Ionic conductivity and viscosity of the various electrolytes at room temperature. (h) Electrolyte wettability on the polyethylene separator. (i) Rader chart comparing the cell performances and physical properties of the electrolytes with various FEC/PC ratios.

In the SIB system, the electrolytes typically rely on 1 M of NaPF_6_ or NaClO_4_ dissolved in single (e.g. PC) or mixed (e.g., ethylene carbonate (EC)/PC or EC/dimethyl carbonate (DMC)) carbonate solvents [19]. To make it simple, single PC-based solvent with 1 M NaPF_6_ was adopted as a prototype electrolyte here, which was then mixed with various ratios of FEC in probing the concentration-dependent Na metal performances. As expected, with 1 M NaPF_6_ in PC (denoted as N-P), the initial CE (ICE) of SMA was only 21.8% in a Na/Cu cell. The CE then experienced severe fluctuations varying from 4% to 24%, followed by consistently low values of approaching 0% in no more than 20 cycles. Interestingly, with 10 vol% of FEC incorporated, the electrolyte of 1 M NaPF_6_ in FEC/PC (1 : 9 by vol) (referred to as N-FEP-19) afforded a significantly higher ICE of 85.6%, denoting the effectiveness of FEC in enhancing the Na reversibility. Nevertheless, the cell was still seen to undergo obvious CE decay after the initial 3 cycles, delivering a quite low average CE of 77.34% for 50 cycles. Upon raising the FEC ratio to 20 vol% in N-FEP-14 (1 M NaPF_6_ in FEC/PC (1 : 4 by vol), the ICE kept rising to 89.3%, yielding an average Na CE of 92.04%. Further addition of FEC to a FEC/PC ratio of 1 : 1 (N-FEP-11), however, did not produce profound CE improvement (93.58%). When substituting all PC with FEC solvent, both the ICE and the average CE reached the highest values of 90.2% and 95.12% in the N-FE electrolyte, respectively (Figure 2(a)). Yet, we also notice the widened voltage polarization at a high FEC concentration, indicating an increased interfacial resistance (Figure S1). This observation was then confirmed by electrochemical impedance spectroscopy (EIS) measurements, where at a lower FEC concentration of 10 vol%, the resistance of Na drops markedly from 731 *Ω* in N-P to 153 *Ω* after 3 cycles in Na/Cu cell configurations (Figure S2). While at FEC concentrations above 20 vol%, the corresponding impedance builds up steadily, reaching 124, 187, and 216 *Ω* in N-FEP-14, N-FEP-11 and N-FE, respectively. However, as Na plating/stripping proceeds to 50 cycles, the impedance spikes to 1159 and 503 *Ω* using N-P and N-FEP-19, whereas the corresponding values using N-FEP-14, N-FEP-11, and N-FE electrolytes are 169, 217, and 263 *Ω*, respectively, which manifest minor increment compared to that after 3 cycles (Figure 2(c)). It can be thus concluded that the FEC-induced SEI can be somewhat ionically impeding after initial formation, but shows great efficiency in stabilizing the interface against subsequent deteriorations, which again ascertains a threshold FEC concentration needy to be established in balancing Na passivation with the interfacial kinetics.

Further verification of the concentration-dependent performance was carried out in Na/Na symmetrical cell configurations, allowing us to investigate the long-term plating/stripping durability of SMAs. As seen in Figure 2(d), the voltage profile of the N-P-based cell showed rapidly enlarged polarization, which then failed after ~80 h as a result of voltage spiking. Such phenomenon is often observed with carbonate-based electrolytes, where unceasing Na/electrolyte reactions occur, causing severe dead Na and SEI accumulation that eventually blocks the ion conductive channels [20–22], whereas 10 vol% of FEC was shown efficacious in prolonging the cyclability to ~400 h. Further addition of FEC resulted in longer-term Na cycling over 500 h without observable voltage rise. While agreeing with the former results with Na/Cu cells, the Na plating/stripping overpotential grows with increased FEC concentrations, and the observation was later corroborated with the EIS and Tafel plot evidences (Figure S3 to and S5).

Finally, we move into full-cell configurations in testifying such concentration-dependency in practical batteries. Na_3_V_2_(PO_4_)_2_O_2_F (NVPOF) is selected as the cathode here for its high voltage plateaus (3.6 and 4.0 V vs. Na^+^/Na) and a high theoretical capacity of 130 mAh g^−1^. During cycling tests at 1 C, the Na/NVPOF cell with the N-P electrolyte experienced fast capacity loss after ~130 cycles, possibly originated from the SEI build-up or the electrolyte depletion [23], delivering a short cycle life of no more than 200 cycles. Changing the electrolyte to N-FEP-19 immensely boosted the cyclability to 500 cycles, suggestive of the positive role of FEC also on the cathode side. Yet, the capacity retention of 84.3% was still unsatisfactory. At FEC ratio above 20 vol%, the capacity retention became similarly high at 93.7%, 93.9%, and 95.8% with the N-FEP-14, N-FEP-11, and N-FE electrolytes, respectively (Figure 2(e), Figure S6). However, this trend failed to sustain for rate tests ranging from 1 C to 20 C. As indicated in Figure 2(f), the best cell performance was afforded with the N-FEP-14 electrolyte, from which the discharge capacities of 110.8, 105.5, and 95.3 mAh g^−1^ were achievable at 10, 15, and 2 C, respectively, setting sharp contrast to the values of 57.3, 34.6, and 21.6 mAh g^−1^ attained with the blank electrolyte. With N-FE electrolyte, however, the rate performance underwent degradation compared to the N-FEP-14 and N-FEP-11 counterparts (discharge capacities of 94.2 and 73.6 mAh g^−1^ at 15 and 20 C), consistent with the higher impedance and polarization seen in the full cells at a high FEC concentration (Figure S7 and S8). Such deteriorated rate performance with high FEC concentration is in agreement with the increased interfacial impedance obtained, suggesting that the SEI film may be too thick with a high concentration of the film-forming solvent added. Moreover, the sacrifice in both the electrolyte conductivity and the wettability (Figures 2(g) and 2(h)) may constitute other kinetic barriers at high rates. Thus, combined with the physical properties of the electrolytes that higher FEC ratio produces higher viscosity, lower ionic conductivity, and poorer wettability for the electrolytes, we would suggest an optimal FEC/PC ratio at 1 : 4 (by vol) where we achieve an overall optimized performance between Na CE, full cell behavior, and the physical properties (Figure 2(i)).

### 2.2. Establishing the Threshold FEC Concentration through Its Coordination State

**Figure 3:**
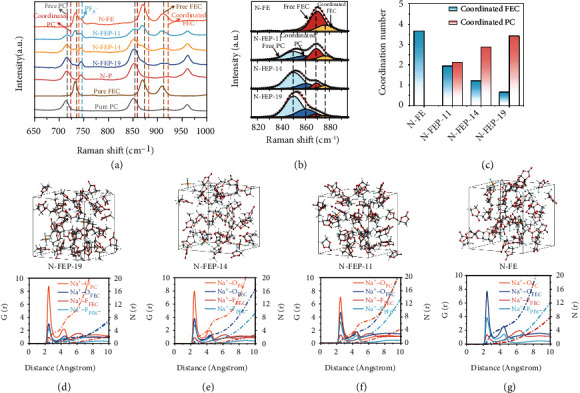
Solvation study of the electrolytes based on various FEC concentrations. (a) Raman spectra of the corresponding solvents and electrolytes with various FEC/PC ratios. (b) Detailed fitting of the Raman spectra of the corresponding electrolyte in a range of 815 to 895 cm^−1^. (c) Bar chart showing the coordination number of FEC and PC solvents in the corresponding electrolyte. Snapshots of the (d) N-FEP-19, (e) N-FEP-14, (f) N-FEP-11, and (g) N-FE electrolytes as extracted from the MD simulations, with the corresponding RDF of the Na^+^-O_PC_, Na^+^-O_FEC_, Na^+^-F_FEC,_ and Na^+^-F_PF6-_ pairs provided below.

The coordination state of the molecules largely dictates their decomposition sequence and thus the contribution to the SEI. On one hand, the coordinated species would travel with cations to priorly reach the anode during Na^+^ plating, winning to decompose priorly on Na surface by a preferential physical contact. On the other, the reduction potential of the molecules manifests an increase upon their coordination with Na^+^, again triggering an early onset of their decomposition electrochemically [24, 25]. Thus, it is vital to investigate the coordination states of FEC at various concentrations, so as to probe its reduction sequence on SMA in the bulk electrolyte. For that, Raman spectroscopy was first conducted on a range of FEC/PC electrolytes, from which we are able to identify the peaks for free PC, free FEC, and the coordinated PC and FEC molecules in each electrolyte (Figure 3(a)). Since the peak intensities of the free FEC and PC molecules differ significantly, it is then meaningless to directly compare the ratios of coordinated FEC or PC molecules by analyzing the peak areas from Raman Spectra. However, we can deconvolute the percentage of coordinated FEC vs. all FEC molecules and the coordinated PC vs. all PC molecules. Then, based on the total molar amount of the FEC and PC molecules present in the corresponding electrolyte, the coordination numbers for FEC and PC can be to quantitatively estimated. The range of 815-895 cm^−1^ was then selected to deconvolute the solvent species from the Raman spectra. As shown in Figure 3(b), the free FEC and PC molecules exhibit bands at 869 and 850 cm^−1^, respectively, whereas peaks at 876.5 and 860.5 cm^−1^ are attributed to their coordinated counterparts. By analyzing the deconvoluted peak areas, it is found that the coordination percentage for FEC molecules is 25.9, 27.5, 43.2, and 47.7% in the N-FE, N-FEP-11, N-FEP-14, and N-FEP-19 electrolytes, respectively, corresponding to a FEC coordination number (CN) of 3.66, 1.94, 1.21, and 0.66, respectively. Similarly, the CN of PC was calculated to be 2.12, 2.88, and 3.41 in N-FEP-11, N-FEP-14, and N-FEP-19, respectively (Figure 3(c)). The lower CN number of FEC even with FEC/PC ratio of 1 : 1 was originated from the poorer solvation power of FEC compared with PC, as stemming from the strong electron withdrawing effect of the F atom attached to FEC [26, 27].

In parallel to the spectroscopy results, we performed molecular dynamic (MD) simulations on the respective electrolytes in providing further insights into the solvation structure. Seen in Figures 3(d)–3(g) are the snapshots of the simulated electrolyte with the radial distribution functions (RDFs) of Na^+^-coordination pairs. Despite the prominent peak assigned to Na^+^-O_PC_ pairs observed at ~2.2 Å for all electrolytes, the results clearly demonstrate the gradually enhanced peak for Na^+^-O_FEC_ pairs with increasing FEC concentrations, indicating the intensified FEC participation into the primary Na^+^ solvation sheath. Such FEC involvement, even in the minority, is important for its contribution to the SEI. Moreover, the signal for Na^+^-F_PF6_^−^ pairs is also seen to strengthen accordingly, which agrees with the relatively poor solvation capability of FEC that results in more anion participation into the solvates. We also notice that the simulated coordination number of FEC and PC well overlaps with our experimental observation (Table S1), indicating the CN of FEC to be ~1.26 at the optimal FEC/PC ratio of 1 : 4.

In demonstrating the CN of FEC as a key indicator, we turn to explore the concentration-dependency of SMAs in ethyl methyl carbonate- (EMC-) based electrolyte, where FEC definitely wins in coordinating with Na^+^ ions over the PC-based one for the much lower solvating power of EMC. As seen in Figure S9, though the CE performance at FEC/EMC ratio of 1 : 1 and 1 : 4 (by vol) was even slightly inferior than the PC-based counterpart, we find the CE decay was largely suppressed with at the ratio of 1 : 9. By looking into the detailed Raman spectra, coordinated FEC accounts for 73.4% in all the FEC molecules, corresponding to a CN value of 1.03, much higher than the CN of FEC achieved in N-FEP-19 (Figure S10). Thus, we speculate the threshold CN for FEC to be close to ~1.3, where minor or no electrochemical enhancement can be expected beyond this value; yet, significantly lowered Na CE is seen below the value.

**Figure 4:**
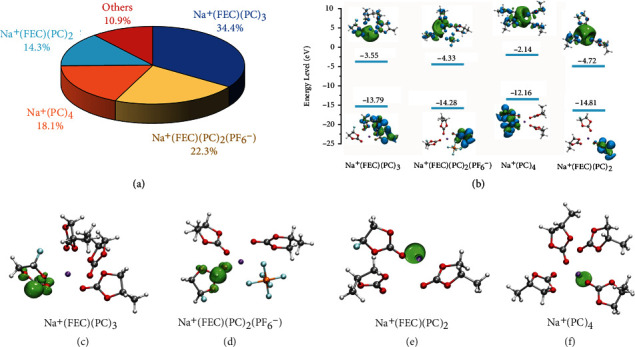
Simulations predicting the decomposition sequences of the solvation clusters. (a) Pie chart illustrating the dominant Na^+^ solvates in the N-FEP-14 electrolyte based on the trajectory data from MD simulation. (b) The HOMO and LUMO energy levels of the Na^+^(FEC)(PC)_3_, Na^+^(FEC)(PC)_2_(PF_6_^−^), Na^+^(PC)_4_, and Na^+^(FEC)(PC)_2_ solvates. Geometries and the spin density mapping of the (c) Na^+^(FEC)(PC)_3_, (d) Na^+^(FEC)(PC)_2_(PF_6_^−^), (e) Na^+^(PC)_4_, and (f) Na^+^(FEC)(PC)_2_ solvates.

To investigate how such threshold FEC concentration works to passivate the Na surface, we further conducted density functional theory (DFT) calculations in studying the reduction tendency of FEC molecules once they form specific complexes with Na^+^. Specifically, based on the trajectory data obtained from MD simulations, we extract four dominant solvates in the N-FEP-14 electrolyte to calculate their LUMO levels (Figure 4(a), Figure S11). Because of the higher CN of PC, the existence of Na^+^(PC)_4_ solvate is also seen to account for a percentage of 18.1%. However, its LUMO level is significantly higher than the FEC-involved counterpart (Figure S12), making it much less prone to reduce on SMAs. On the other hand, LUMO levels of the FEC-involved solvates manifest obviously lower values comparing to the free solvent molecules, in consistence with the previous literatures, which enables them to primarily affect the SEI due to their lowest LUMO level of -4.72 and -4.33 eV, respectively (Figure 4(b)). Spin density analysis was thus conducted on the four dominant clusters in elucidating the sites vulnerable to accept electrons, as mapped in the green-colored areas (Figures 4(c)–4(f)). With FEC involving into the solvation sheath, the spin electron (as represented by the isosurface in green) is found to either reside on its O=CO(O) moiety, or sharing between Na^+^ and its C=O bond, indicating the FEC molecules to decompose preferentially among the other solvation species by its higher tendency to accept the extra electrons [28]. Such that a rapid passivation on SMAs can be initiated by FEC molecules at a higher potential when PC and its derivates are not yet to decompose. Noteworthy, with FEC-involved solvates reducing priorly, the bulk equilibrium would then shift to maintain their population by pushing uncoordinated FEC into the solvation sheaths, thus sustaining the reactions [29].

### 2.3. Morphological and Compositional Analysis of SMAs with FEC Cosolvation

**Figure 5:**
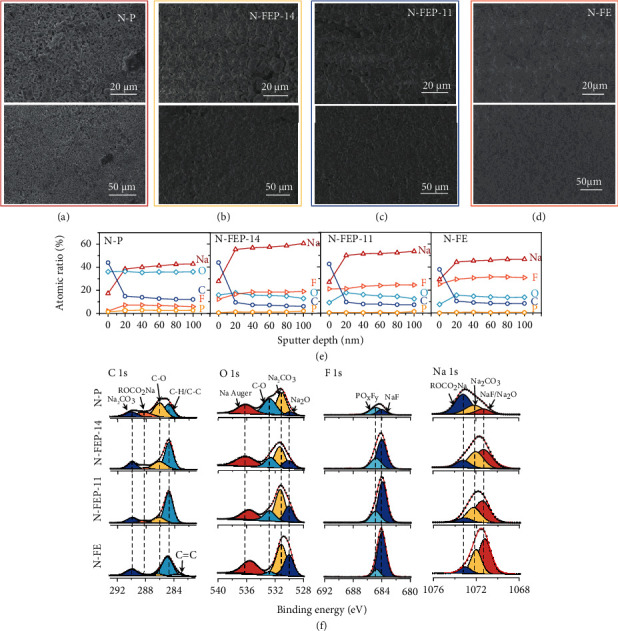
Postmortem SEM and XPS characterizations on SMAs. SEM images of the deposited Na using the (a) N-P, (b) N-FEP-14, (c) N-FEP-11, and (d) N-FE electrolytes at various magnifications with a plating amount of 2.0 mA/cm^2^ at 0.5 mA/cm^2^. (e) Atomic ratio of the C, O, F, and Na element in the SEI at various sputtering depths in the corresponding electrolyte. (f) C 1 s, O 1 s, F 1 s, and Na 1 s XPS spectra of the SEI on Na metal formed in the corresponding electrolyte.

In deepening into the causes for the drastic CE improvement above the threshold FEC concentration, Na deposition morphologies are firstly imaged in the corresponding electrolytes to corroborate the electrochemical evidences. Shown in Figures 5(a)–5(d) are the scanning electron microscopy (SEM) images of the electrodeposited Na on Cu foil at 0.5 mA/cm^2^, with the digital pictures depicted in Figure S13. Using the FEC-free N-P electrolyte, typical Na dendrites are observed with a loose and highly tortuous structure, which exponentially increases the Na exposure area and puts large hinderance to subsequent Na stripping. With FEC added, Na deposits are seen to grow in considerably larger size, rendering an enhanced packing density that minimizes the contact area between Na and the electrolyte. Such decreased tortuosity of Na also helps to maintain its structural connection from the tip to the base, guaranteeing a more thorough Na stripping and thus the much higher ICE [30, 31]. By excluding all PC molecules, Na deposition shows the largest packing density in N-FE, in accordance with the highest ICE obtained. The results somehow reflect that, though with PC decomposition largely suppressed by FEC-involved solvates, corrosion from PC is still inevitable when it exists in the electrolyte. Such observations are further verified by the post-mortem SEM images shown in Figure S14, where the metallic Na retrieved from pure PC-based N-P electrolyte shows a porous structure with pervasive curvatures. Whilst the cosolvation of FEC was shown to tremendously inhibit such corrosion, where smooth Na morphologies are observed especially at higher FEC concentrations.

For the dictating role of FEC-involved solvates in affecting the SEI, indepth X-ray photoelectron spectroscopy (XPS) profiling was thus performed at various FEC concentrations. As seen in the elemental distribution at different SEI depths, without FEC, minor existence of *F* was detected from the surface to the bottom (~2 at% and~5.78 at%, respectively), which is understandable since *F* source only comes from the anions here (Figure 5(e), Figure S15). We instead detected the apparent enrichment in C and O signals on the surface, with their deep penetration throughout the SEI, confirming the severe electrolyte attack taken place [32, 33]. With FEC, steadily rising *F* ratio is observed as FEC concentration increases (from ~12.18 at% in N-FEP-14 to ~25.19 at% in N-FE), since more *F* sources are incorporated accordingly. Meanwhile, the O element takes up smaller concentration of ~10-20 at%. And C element, though still dominant in the outer SEI, experiences sharper decrease upon sputtering comparing to that using the N-P electrolyte. Notably, the atomic percentage of Na element gradually strengthens with sputtering depths, suggesting that we are approaching the Na surface. However, from the N-FEP-14 electrolyte to N-FE, the overall Na ratio is seen to decline, which may suggest a thicker SEI formed at higher FEC concentrations. But above all, Na ratio of the SEI induced in FEC-containing electrolyte all manifests higher value than that of the control sample.

We then turn to high-resolution XPS spectra where detailed SEI species can be fitted accordingly (Figure 5(f)). The identified chemistries generally include the organic moieties of C-C/C-H, C-O and ROCO_2_Na and the inorganic components such as Na_2_CO_3_, Na_2_O, NaF, and PO*_x_*F*_y_* [3, 16, 34]. Unexpectedly, the incorporation of FEC did not bring new species into the SEI, in contradictory with some works reporting the generation of poly (vinylene carbonate) or -CHF-OCO_2_- (PEO) with FEC polymerization on silicon anodes [14, 35]. However, we are able to differentiate the obvious changes in the relative intensities between each species. Comparing with the N-P-derived SEI, a prominent distinction with the FEC-containing electrolytes comes from the tremendously decreased organic species of -C-O- and ROCO_2_Na. Instead, FEC leads to a SEI with intensified contribution from the inorganics, specifically the NaF and Na_2_O, and this tendency grows with increasing FEC concentration, which complies with the calculation results that coordinated FEC undergoes fast defluorination with almost no activation barrier [29, 36]. Moreover, the lower atomic ratio of P element in the FEC-containing electrolytes, accompanied by the gradually decreasing PO*_x_*F*_y_*/NaF proportion ratio in the F 1 s spectrum, implies the inhibited decomposition of PF_6_^−^ as FEC increases. The enrichment in other inorganics can be explained by the common-ion effect, through which a saturated solution of NaF lowers the free-energy barrier for other Na-containing particles to precipitate, inducing more Na_2_CO_3_ and Na_2_O components [35].

### 2.4. Cryo-TEM Imaging on the Microstructure of the Na Deposits and the SEI

**Figure 6:**
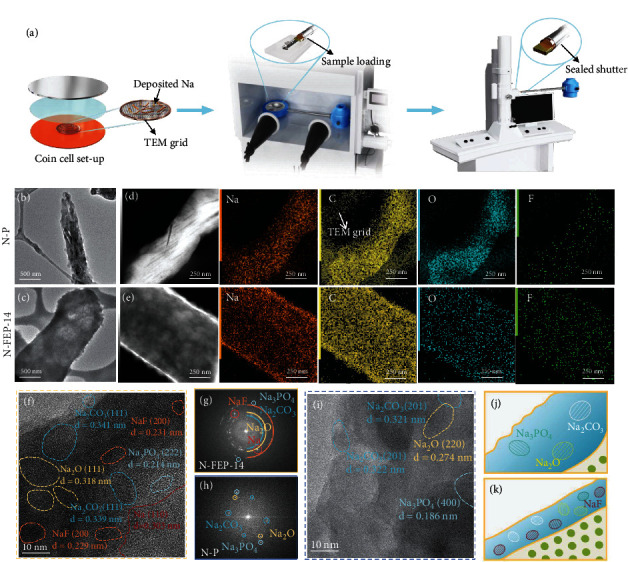
Morphology of a single Na filament and its SEI nanostructure with and without FEC cosolvation. (a) Schematic illustration on the sample preparation and transfer processes for cryo-TEM detection of the deposited Na. The deposited Na filament on TEM grid using the (b) N-P and (c) N-FEP-14 electrolyte. Cryo-STEM DF image with the corresponding elemental mapping of the deposited Na in the (d) N-P and (e) N-FEP-14 electrolyte. HRTEM images showing the SEI composition on Na metal formed in the (f) N-FEP-14 and (i) N-P electrolyte, with the corresponding FFT images shown in (g) and (h). Schematics showing the Na SEI structure formed in (j) N-P and (k) N-FEP-14.

Despite of the chemical compositions, the SEI structure also plays a decisive role in dictating the cell performances. However, traditional XPS measurements fall short in providing high-spatial resolution of the arrangement of these species in the SEI. Fortunately, the emerging Cryo-TEM technique makes the capture of individual Na filament and the atomic-resolution characterization on its SEI possible. As schematically shown in Figure 6(a), Na deposits are firstly electroplated onto a Cu TEM grid at 0.5 mA/cm^2^ and 1.0 mAh/cm^2^ using the coin-cell configuration, after which we extract the grid and load it onto the cryovacuum-transfer holder in Ar-filled glovebox. The specially designed sealing shutter guarantees an all-time protection in Ar atmosphere, where the ingress of air, liquid N_2_, or any other contaminants is avoided during the sample transfer. After the holder was inserted into the TEM chamber, cooling was realized by adding liquid N_2_ in the dewar of the holder that allows us to capture the sample at around -178°C. Figures 6(b) and 6(c) compare the individual Na filaments plated using the blank N-P electrolyte and the N-FEP-14 electrolyte where we achieve the optimal cell performance. One major distinction comes from the deposition size; without FEC, Na exhibits a typical dendrite structure with long aspect ratio, possessing a thin diameter of ~400 nm. By contrast, the N-FEP-14 electrolyte facilitated a profoundly thickened Na to a diameter of >1 *μ*m, which corroborates with our SEM results (Figures 4(a) and 4(b)): another distinction stems from the surface conditions of the deposited Na. In the N-P electrolyte, the contrast of bulk Na darkens, indicating its complete wrapping with a thick SEI layer. Whilst using N-FEP-14, the Na filament manifests a profoundly lighter contrast, with uniform and explicit edges seen on the boundary. This observation is better verified using the cryo-STEM dark-field (DF), where we can spot an obvious color contrast between bulk Na and the SEI layer. As seen in Figure 6(e), a uniform SEI layer (white color) emerges with a thickness of ~40 nm along the edge of Na, in contrast to the all white-colored Na using the corrosive all-PC electrolyte (Figure 6(d)). Further evidence from elemental mapping reveals the hybrid SEI containing prominent Na, C, O, and F signals, with the FEC-added experiencing a decreased C intensity along with an increased F intensity, in line with the XPS observations.

Cryohigh-resolution TEM (cryo-HRTEM) images present the SEI from both electrolytes to be moisaic where the inorganics coexist with organics, while the N-FEP-14-induced SEI shows obviously more nanosized crystalline domains (Figures 6(f) and 6(i)). Combined with the strong ring patterns from the fast Fourier transform (FFT) image shown in Figure 6(g), we identify the highly crystalline nature of the SEI derived with FEC. As marked in Figure 6(f), a crystallized Na metal domain is resolved with the lattice spacing correlating to the (110) plane, above which lies the SEI region with rich and randomly distributed nanocrystals, indexing either to Na_2_CO_3_, Na_2_O, NaF, or minor Na_3_PO_4_. Despite the all-known benefits brought by NaF, such inorganic-rich SEI is particularly attractive since inorganics are found to be more efficient for electronic-insulation and ionic-conduction in the SEI. In terms of Na^+^ transport, inorganic components species tend to work synergistically in increasing ionic carrier concentration between their grain boundaries, providing facile transport channels for cations to migrate through [37–39]. This explains the reason for the drastically lowered Na impedance upon adding FEC. The SEI configuration formed in N-P electrolyte stands a stark contrast to that emerges upon FEC cosolvation, where a nonuniform and largely amorphous layer is seen with only the amorphous matrix with only sparse nanocrystals (Figure 6(i)), affording a weak FFT pattern accordingly. Though *F* signal was captured from the mapping results (Figure 6(h)), its absence in the HRTEM image may imply its existence at very low concentration. Such organic-rich SEI is unfavorable in passivating the hyperreactive anodes like Na metal. Since organics are usually porous in nature, they often fall short in surviving the electrolyte permeation, thus giving rise to a sustained electrolyte consumption and the rapid impedance spike observed in Figure 1(c) [40, 41]. More importantly, as resulted from the rich inorganic species, the FEC-derived SEI manifests a significantly higher average Young's modulus (11.5 GPa) than its PC counterpart (7.3 GPa), representing an enhanced capability in suppressing the dendrite evolution by physical blocking (Figure S16).

### 2.5. Universality of the Cosolvation Strategy

Based on the above rationale, the cosolvation of FEC was further investigated with a serious of the “corrosive solvents,” whose application in SMBs has also been largely hindered by their severe parasitic reactions with Na metal. Specifically, three solvents, namely, EA, TMP, and AN, were chosen for their superior functions in fast-charging, fire-extinguishing and high-voltage charging, respectively. As demonstrated in Figure S17 to Figure S19, the incorporation of FEC is seen to provide drastic increase for the Na plating/stripping CE (from ~5-30% to >92%) in EA, TMP, and AN-based electrolytes with 1 M NaPF_6_. Notably, similarly high Na CE was obtained at specific FEC concentrations despite of the main solvent used. Such observation indicates that the FEC-induced SEI to dominate Na plating/stripping upon its cosolvation with other corrosive molecules, whilst shielding away the corrosive solvent molecules, which manifests a remarkable advancement to extend the choice of the electrolyte recipes in SMBs. As in SIBs, binary carbonate solvents (e.g. EC/PC) are often employed, we thus furthered the evaluation of FEC cosolvation with the binary EC/PC-based electrolytes. As shown in Figure S20, the cosolvation of EC in PC-based electrolyte failed to provide enhancement to the Na reversibility, where a low average CE of 13.57% was delivered in 1 M NaPF_6_-EC/PC (1 : 1 by vol) electrolyte. While with 20 vol% of FEC involved, the reversibility increases substantially with an average CE value of 93.07% for 50 cycles. Such extension for binary solvent-based electrolytes is critical as more than one solvent is often simultaneously incorporated in the electrolyte recipe for their complimentary effects.

## 3. Conclusion

In summary, via a FEC cosolvation strategy, we enable highly reversible sodium metal anodes with the conventional carbonate-based electrolytes, significantly boosting the Na CE from ~20% to ~95%. Through systematic investigations on both the electrochemical performances and physiochemical properties, a threshold FEC concentration is established as related to a coordination number of ~1.2, where adequate reduction from the FEC-involved solvates can be guaranteed to preferentially passivate Na metal at above this value. Further, we show that FEC renders a considerably larger Na deposit size, promoting a more thorough Na stripping while reducing the Na/electrolyte contact areas. Cryo-TEM identifies a thin, highly crystalline, and inorganic-rich SEI formed with FEC, distinct from the thick and largely amorphous SEI observed in the control electrolyte. Such unique nanostructure not only facilitates a facile Na^+^ transfer but also endows the SEI with high mechanical strength, effectively leading to the long-term Na metal cyclability. The protocol established herein was also extendable using other “corrosive solvents” including EA, TMP, AN, or the binary carbonate solvents (e.g., EC/PC), allowing us to surpass the Na metal corrosion brought by the corrosive solvent molecules, thus bringing opportunities for the application of a variety of solvents towards highly reversible SMAs.

## Supplementary Materials

Supplementary Materials The detailed experimental methods on electrolyte preparation, characterizations, calculation methods, and electrochemical measurements, and Figures S1-S20 are incorporated in the supplementary material.
