# Supplementary material for: Deciphering the Role of Fluoroethylene Carbonate towards Highly Reversible Sodium Metal Anodes
Source: Research (Wash D C). 2022 Jan 27;2022:9754612. doi: 10.34133/2022/9754612 (PMC8817186; doi:10.34133/2022/9754612)
Supplement: Supplementary Materials — The detailed experimental methods on electrolyte preparation, characterizations, calculation methods, and electrochemical measurements, and Figures S1-S20 are incorporated in the supplementary material. [file 9754612.f1.doc]

*Supporting Information for:*

**Deciphering the role of fluoroethylene carbonate towards highly reversible sodium metal anodes**

Xueying Zheng,^1^ Suting Weng,^2^ Wei Luo,^1^,* Bo Chen,^1,3^* Xiao Zhang,^2^ Zhenyi Gu,^4^ Haotian Wang,^1^ Xiaolu Ye,^1^ Xuyang Liu,^1^ Liqiang Huang,^1^ Xinglong Wu,^4^ Xuefeng Wang,^2,5^,* Yunhui Huang^6^,*

^1^Institute of New Energy for Vehicles, Shanghai Key Laboratory of Development & Application for Metallic Functional Materials, School of Materials Science and Engineering, Tongji University, Shanghai 201804, China

E-mail: [weiluo@tongji.edu.cn](mailto:weiluo@tongji.edu.cn)

^2^Institute of Physics, Chinese Academy of Sciences; School of Physical Sciences, University of Chinese Academy of Sciences, Beijing 100190, China

E-mail: [wxf@iphy.ac.cn](mailto:wxf@iphy.ac.cn)

^3^Key Laboratory of Advanced Civil Engineering Materials (Tongji University), Ministry of Education, Shanghai 201804, China

E-mail: bo.chen@tongji.edu.cn

^4^Key Laboratory for UV Light-Emitting Materials and Technology, Northeast Normal University, Ministry of Education, Changchun, Jilin 130024, China

^5^Tianmu Lake Institute of Advanced Energy Storage Technologies Co. Ltd., Liyang, Jiangsu 213300, China

^6^State Key Laboratory of Materials Processing and Die & Mould Technology, School of Materials Science and Engineering, Huazhong University of Science and Technology, Wuhan, Hubei 430074, China

E-mail: [huangyh@hust.edu.cn](mailto:huangyh@hust.edu.cn)

**Keywords**: Sodium metal batteries; Fluoroethylene carbonate; Solvation regulation; Cryo-TEM imaging; Inorganic-rich SEI

**EXPERIMENTAL PROCEDURES**

**Electrolyte Preparation**

Battery-grade NaPF_6_ salt, and the FEC, EC, PC, EMC and EA solvents were obtained from Duoduo chemical reagent Co., LTD. TMP and AN (purity >= 99.9%) solvents were purchased from Sigma-aldrich. The Na salt was dried at 100ºC overnight in the vacuum chamber attached to the glovebox (MIKROUNA). The solvents were all dried with cleaned molecular sieves (4 Å) for 2 days. The electrolyte preparation was thus conducted in the glovebox (H_2_O and O_2_ content < 0.1 ppm) for the corresponding salt/solvent systems.

**Electrochemical Measurements**

The 2032-type coin cells are fabricated to test the electrochemical performance of Na metal anodes using the corresponding electrolytes. In each cell, a glass fiber separator (Whatman GF/D) is used with 160 μL of electrolyte added. For Na/Cu cells, Cu foils were punched into 16 mm disks, while Na metal electrode had a diameter of 12 mm, which were then used to evaluate Na plating/stripping efficiencies. Two symmetric Na metal foils (12 mm) were employed in Na/Na symmetrical cells in measuring the long-term stability of Na metal. Full cells were assembled using Na metal anode pairing with the NVPOF cathode, where the anode and the cathode were punched into diameters of 13 mm and 12 mm, respectively. The full cells were cycled at a voltage range of 2.0-4.3 V versus Na^+^/Na. Fabrication of the NVPOF cathodes can be found in our previous works.^1^ The cycling and rate performance of the cells were all tested using a standard battery tester (Neware, CT4008). Whereas the EIS and CV measurements were performed on the Biologic VMP-3 multi-channel workstation. Specifically, EIS spectra were acquired within a frequency range of 100 mHZ to 10^3^ kHZ, and CV curves were obtained in Na/Na symmetric cell from -0.2 to 0.2 V.

**Characterizations**

Conductivity of the electrolytes were assessed using the conductivity measuring meter (DDS-307, Leici, China), and the viscosity was tested on Thermo HAAKE MARS 60 with a rotator of 60 mm in diameter. Electrolyte wettability was evaluated by capturing their contact angles on the separators via Dataphysics OCA50AF. Raman analysis of the corresponding electrolytes were performed on the LabRAM HR Evolution instrument using a 632.8 nm excitation laser. SEM images of the deposited Na were recorded using Zeiss Sigma 300VP at 5.0 kV. XPS characterizations (American Thermo Fisher Scientific ESCALAB 250Xi) were employed to identify the SEI components formed on Na metal, where an Ar-filled transfer vessel is used to load Na samples in avoiding potential air or moisture contaminations. For Cryo-TEM measurements, as schematically shown in Figure 6A in the manuscript, Na deposits are firstly electroplated onto a Cu TEM grid using the coin-cell configuration, after which we extract the grid and load it onto the cryo vacuum-transfer holder (Fischione 2555) in Ar-filled glovebox. The specially-designed sealing shutter guarantees an all-time protection in Ar atmosphere, where the ingress of air, liquid N_2_ or any other contaminants are avoided during the sample transfer. After the holder inserted into the TEM chamber, cooling was realized by adding liquid N_2_ in the dewar of the holder that allows us to capture the sample at around -178ºC. Cryo-images were acquired using microscope (JEOL JEM F200) under cryogenic temperatures (-178ºC) at 200 kV. All images were analyzed by Digital Micrograph (Gatan) software. The Young’s modulus of SEI film was obtained by PeakForce QNM mode (Bruker Multimode 8) with the RTESPA-525 tip.The retrieved Na metal electrodes were washed thoroughly using PC solvent and dried in the vacuum-chamber attached to the glovebox.

**Calculation Methods**

MD simulations of the Na-ion electrolyte systems were conducted using the Gromacs program suite with an all-atom optimized potentials for liquid simulations (OPLS-AA) force field.^2^ The OPLS-2009IL force field parameters of the PF_6_^-^ anion were acquired from literatures. A charge scaling of 0.8 was applied to mimic the charge transfer and the polarization effects.^3, 4^ The force field parameters of the PC and FEC molecules were generated on the LigParGen web server.^5^ Initial simulation box was fixed at a dimension of 60×60×60 Å^3^ packed with the corresponding electrolyte compositions, which was constructed with the packmol program.^6^ The structures were firstly relaxed with an energy minimizing calculation, followed by an annealing process by elevating the system temperature from 0 to 400 K and further cooled to 298.15 K with a time step of 1 ps during the 2 ns to reach the equilibrium state. Velocity-rescale thermostat was applied to regulate the temperatures at 298.15 K and 243.15 K with a relaxation constant of 1 ps, respectively. To control a pressure at 1.01325×10^5^ Pa, Berendsen's barostat was adopted with an isothermal compressibility constant of 4.5×10^-5^.^7^ Periodic boundary conditions were employed in all directions. To treat the van der Waals forces and the electrostatic interactions, particle-mesh Ewald (PME) method was adopted with a cut-off distance of 10 Å.^8^ After that the energy minimizing calculations and heat equilibrium processes were performed at constant NPT ensemble with the same simulation parameters as above. The MD simulation was then conducted for a total simulation time of 20 ns, with the trajectory data saved for every 1 ps. Further radial distribution functions (RDF) analysis of the electrolyte systems were realized using the trajectory data by Gromacs tool-suites and the Visual Molecular Dynamic program (VMD).^9^

The cluster search was conducted using the python scripts written by ourselves, where the four dominant clusters were chosen to calculate their HOMO and LUMO energy levels. The configurations of the solvent molecules were optimized under the framework of density of functional theory (DFT) with B3LYP functional^10-12^ and 6-31g* basis set.^13, 14^ An explicit sodium ion was added in each simulation system to describe the solvation effect. The implicit solvent model of SMD (Solvation Model Based on Density)^15^ was also applied to correct the solvation effect, and the relative dielectric constants of these mixed electrolyte solvents system were obtained from the MD simulations. In order to obtain the electron structures with higher accuracy, a single point calculation for the optimized structure with M062x functional^16^ and def2TZVP basis set^17^ has been carried out. The spin electron populations were obtained by wavefunction analysis using Multiwfn program.


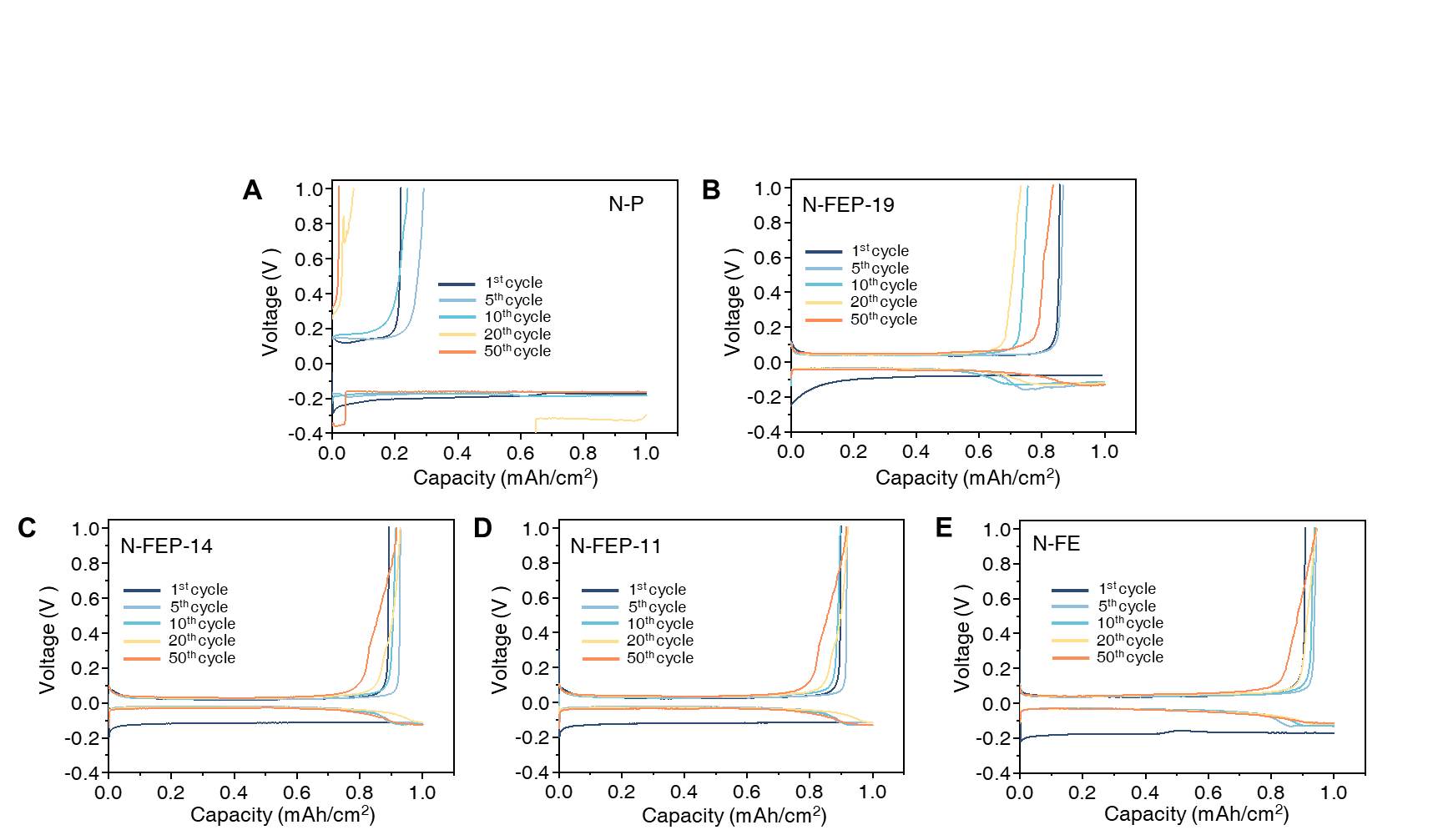


**Figure S1.** The corresponding Na plating/stripping voltage profiles on Cu electrode with (A) N-P, (B) N-FEP-19, (C) N-FEP-14, (D) N-FEP-11 and the (E) N-FE electrolytes at 0.5 mA/cm^2^, 1.0 mAh/cm^2^.

Notably, the voltage hyresteresis decreases with addition of FEC from 10 vol% to 20 vol%, whereas further increasing the FEC concentration beyond 20 vol% resulted in the evidently widened cell polarizations.


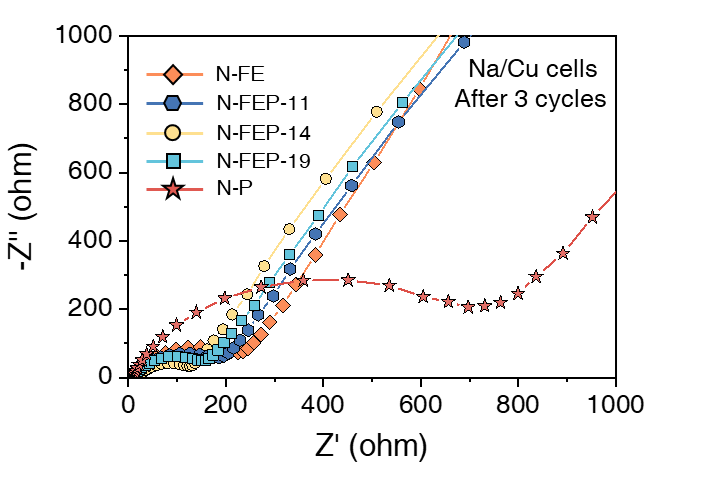


**Figure S2.** Nyquist plots of the Na/Cu cells after 3 cycles (0.5 mA/cm^2^, 1.0 mAh/cm^2^) using the corresponding electrolytes.


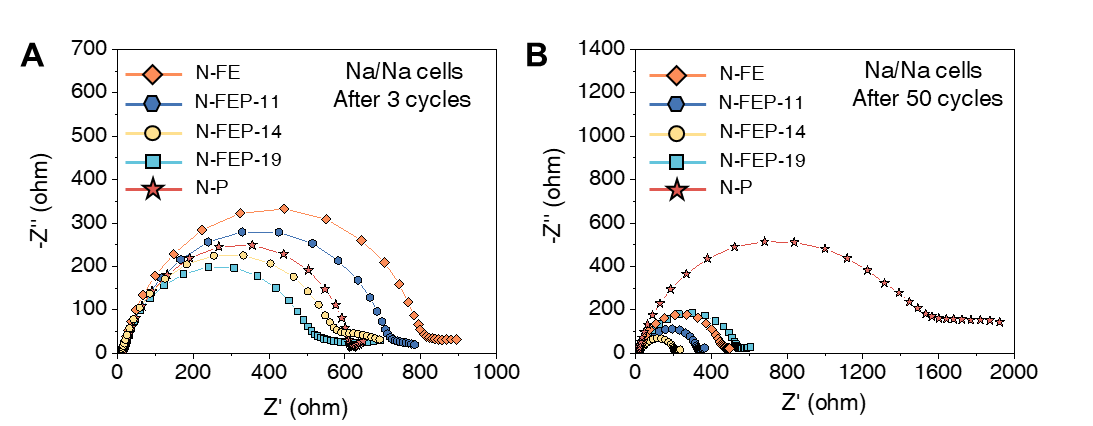


**Figure S3.** Nyquist plots of the Na/Na symmetrical cells after (A) 3 cycles and (B) 50 cycles (1.0 mA/cm^2^, 1.0 mAh/cm^2^) using the corresponding electrolytes.

The incorporation of 10 vol% of FEC in Na/Na symmetrical cells resulted in a massively decreased cell impedance from ~810 Ω to ~500 Ω. However, further addition of FEC beyond 10 vol% led to the gradually increased cell impedance with increasing FEC concentrations, which implies a more ionically-impeding interface formed. However, as cycling proceeds to 50 cycles, the impedance of the cell using blank electrolyte experienced a sharp rise to ~1600 Ω, agreeing with the spiking voltage hysteresis seen in Figure 1d. Whereas the cell with 10 vol% FEC shows quite consistent impedance after cycling. By contrast, at FEC concentration of 20 vol% and above, though the initial impedance was higher, their values experienced a decrease after multiple cycles, suggesting the gradually improved SEI and its ability in suppressing the subsequent electrolyte corrosions.


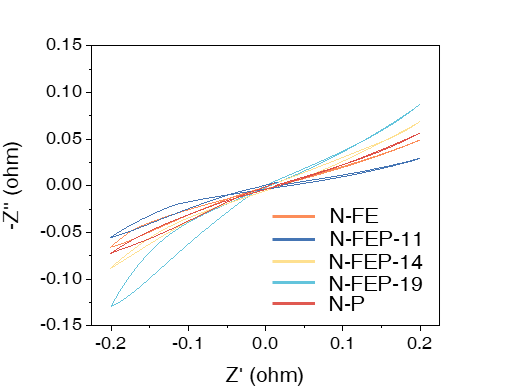


**Figure S4.** Cyclic voltammetry **(**CV) curves of Na/Na symmetric cells with the corresponding electrolyte scanned at 5 mV/s.

The CV profiles were thus used to obtain the Tafel plots. Specifically, the exchange current densities (i_0_) of the Na/Na symmetrical cells are calculated by fitting the cyclic voltammetry profiles into the Tafel equation:
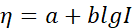
; where
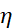
, I represent the overpotential value and current density, respectively, a and b stand for Tafel constant.


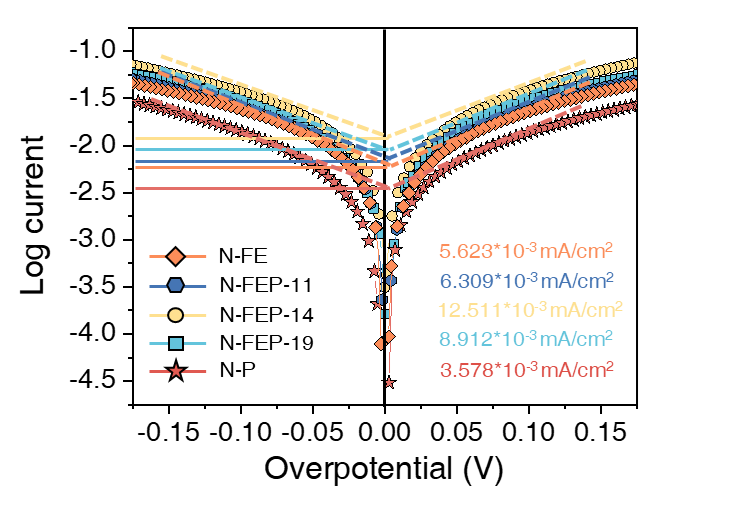


**Figure S5.** Tafel plots for Na plating/stripping in Na/Na symmetrical cells using the corresponding electrolytes. The calculated exchange current densities are shown on the right side as marked in their corresponding colors.

Quite consistent with the EIS evidence in symmetrical cells, the i_0_ values underwent increase from 3.578*10^-3^ to 8.912*10^-3^ and 12.511*10^-3^ mA/cm^2^ with 10 vol% and 20 vol% of FEC added, respectively, denoting the facilitated Na^+^ transfer through the SEI formed in N-FEP-19 and N-FEP-14 electrolytes. Nevertheless, the i_0_ values decreased steadily to 6.309*10^-3^ and 5.623*10^-3^ mA/cm^2^ using N-FEP-11 and N-FE, respectively, suggesting the slowed Na^+^ plating/stripping kinetics at the interface.


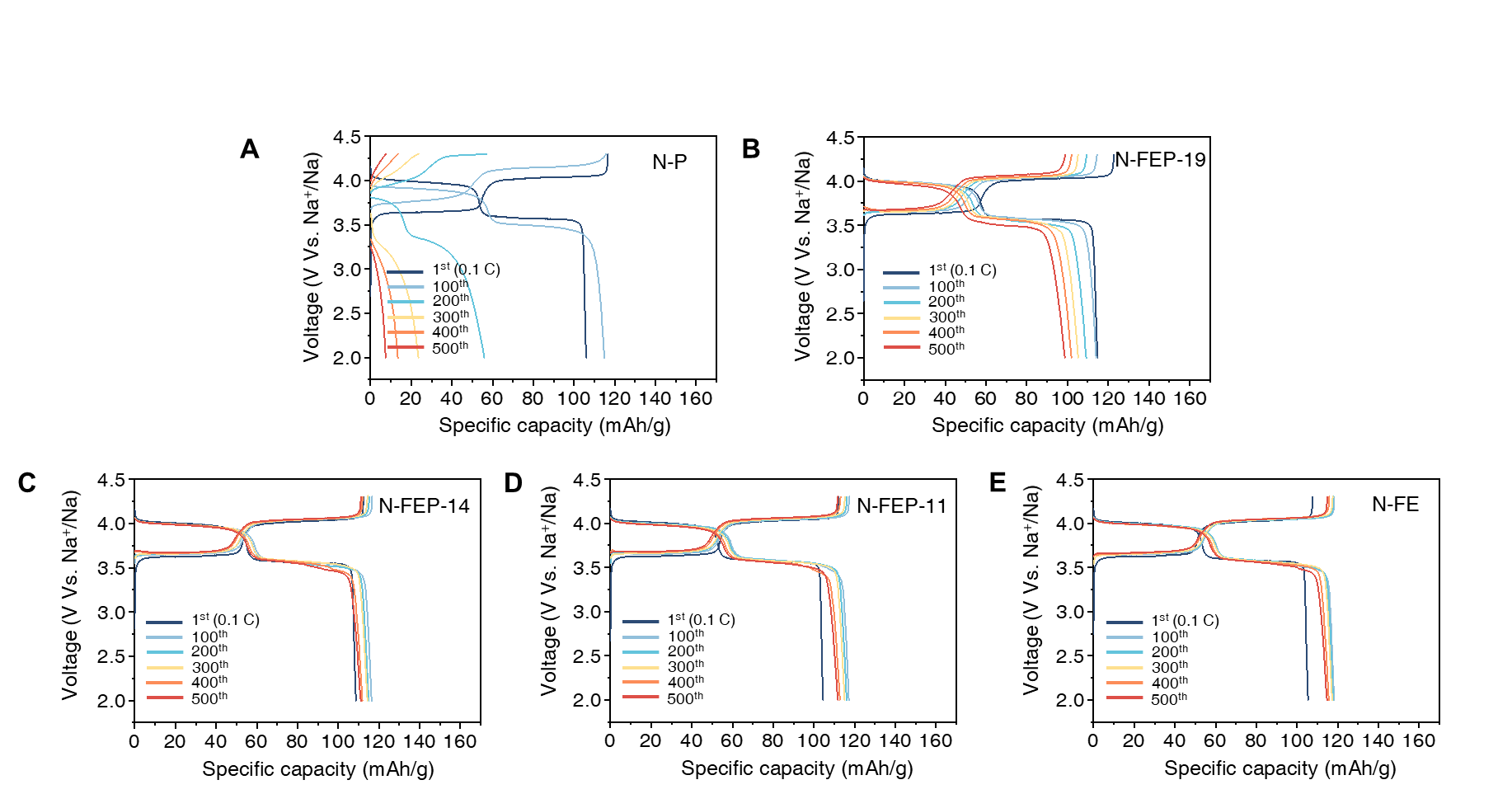


**Figure S6.** Voltage profiles of the Na/NVPOF full cells at increasing rates from 1 C to 20 C using the (A) N-P, (B) N-FEP-19, (C) N-FEP-14, (D) N-FEP-11 and the (E) N-FE electrolytes. Three formation cycles were performed for film formation at 0.1 C.


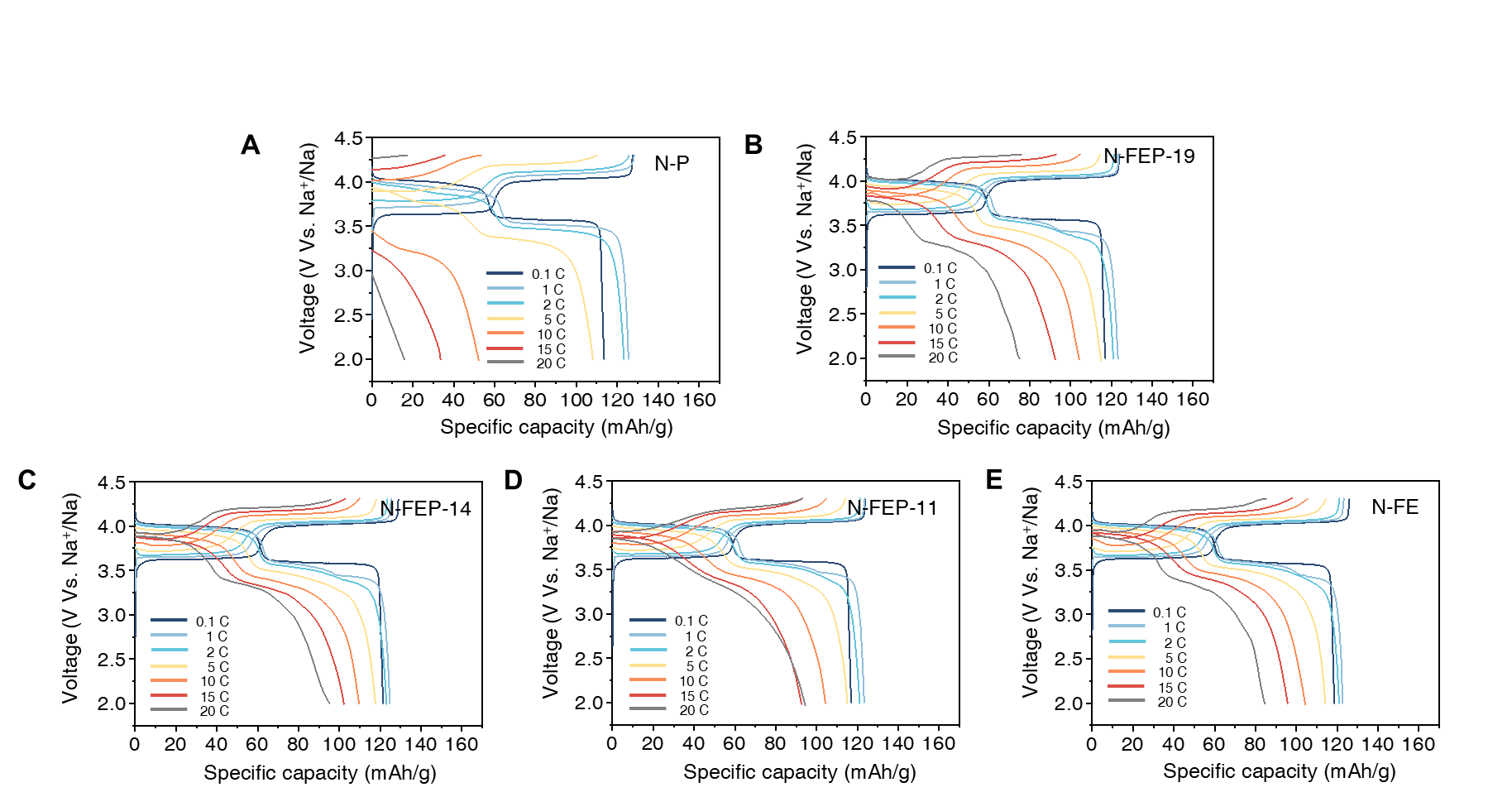


**Figure S7.** Voltage profiles of the Na/NVPOF full cells at the corresponding cycles using the (A) N-P, (B) N-FEP-19, (C) N-FEP-14, (D) N-FEP-11 and the (E) N-FE electrolytes under the rate of 1 C. Three formation cycles were performed for film formation at 0.1 C. Three formation cycles were performed for film formation at 0.1 C.


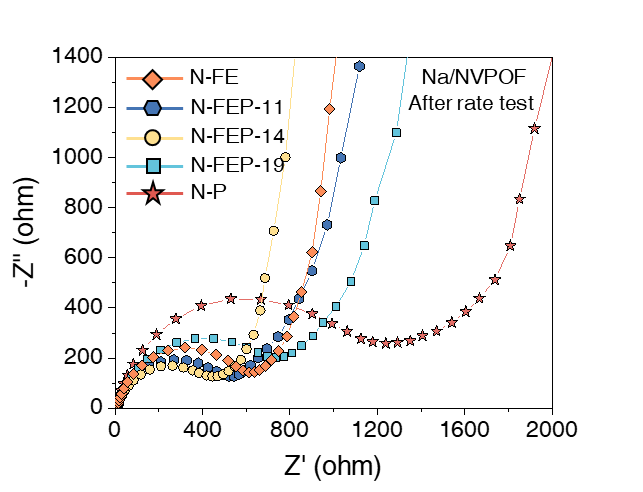


**Figure S8.** Nyquist plots of the Na/NVPOF full cells after rate test using the corresponding electrolytes.

Compared with the FEC-free electrolyte, the resistance of the FEC-added counterparts got decreased obviously, confirming the beneficial role of FEC also on the cathode side. Of all the FEC/PC ratios, the N-FEP-14 electrolyte afforded the lowest impedance, followed by the N-FEP-11, N-FE and the N-FEP-19 electrolytes.

**Table S1.** The calculated coordination number of the corresponding solvent molecules or anions in the 1.0-EP and the 0.8-FEH electrolyte.

| **Electrolyte** | **Coordination number** | | |
| --- | --- | --- | --- |
|  | FEC | PC | PF_6_^-^ |
| N-FE | 3.783 | - | 0.988 |
| N-FEP-11 | 1.893 | 2.357 | 0.694 |
| N-FEP-14 | 1.259 | 2.901 | 0.439 |
| N-FEP-19 | 0.71 | 3.412 | 0.385 |


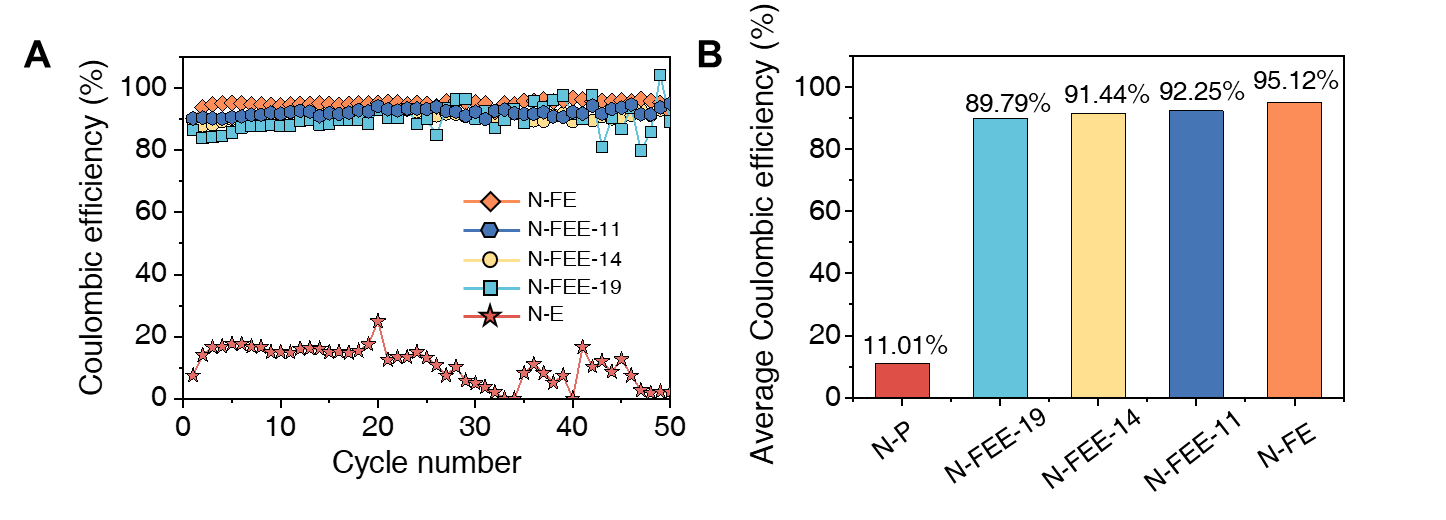


**Figure S9.** (A) The CE for Na plating/stripping assessed in Na/Cu cells using 1.0 M NaPF_6_ in FEC/EMC binary solvents (denoted as N-FEE) at the corresponding volumetric ratios. The cycling condition was 0.5 mA/cm^2^, 1.0 mAh/cm^2^. (B) The calculated average Na CE in the corresponding electrolyte.


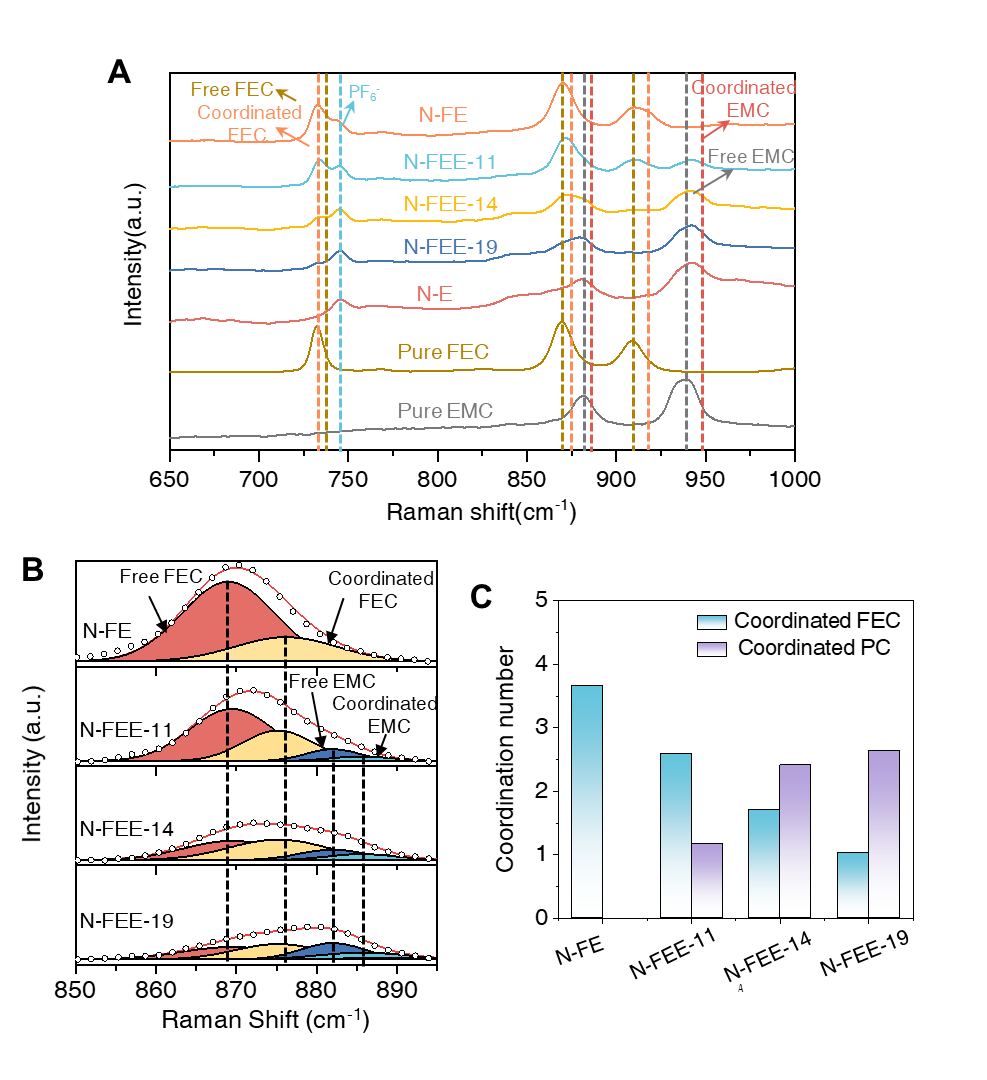


**Figure S10.** (A) Raman spectra of the corresponding electrolyte systems. (B) Deconvoluted Raman peaks the free FEC, free EMC and the coordinated FEC and coordinated EMC species from 850 to 895 cm^-1^. (C) The coordination number of FEC and EMC molecules in the corresponding electrolyte.

Based on the total number of the FEC and EMC molecules present in the corresponding electrolyte, we are able to calculate the coordination number of FEC and EMC by analyzing the percentage of coordinated FEC or EMC molecules versus the total FEC or EMC molecules, respectively.


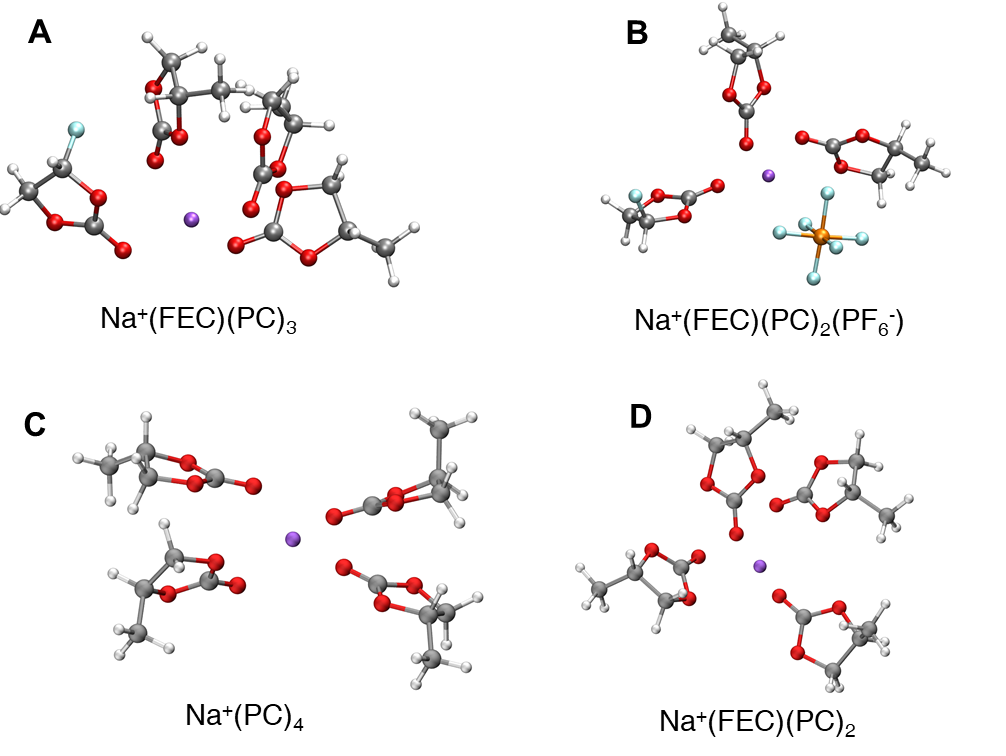


**Figure S11.** The optimized geometrical configuration of the four dominant Na solvates derived from the MD simulations of the N-FEP-14 electrolytes, namely the (A) Na^+^(FEC)(PC)_3_, (B) Na^+^(FEC)(PC)_2_(PF_6_^-^), (C) Na^+^(PC)_4_ and (D) Na^+^(FEC)(PC)_2_. The Na, C, O, H, F and P atoms were marked with purple, grey, red, white, cyan and orange color, respectively.


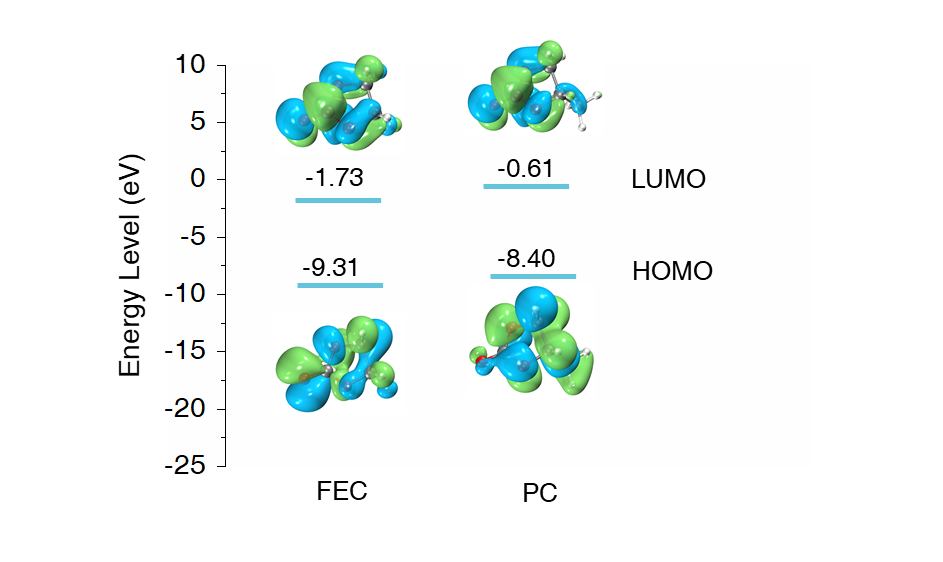


**Figure S12.** The LUMO and HOMO energy levels of the free FEC and PC molecules.


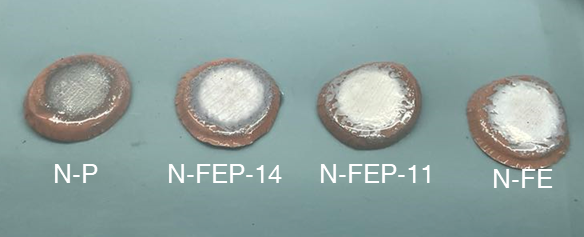


**Figure 13.** Digital photos of the deposited Na using the corresponding with a with a plating amount of 2.0 mA/cm^2^ at 0.5 mA/cm^2^.


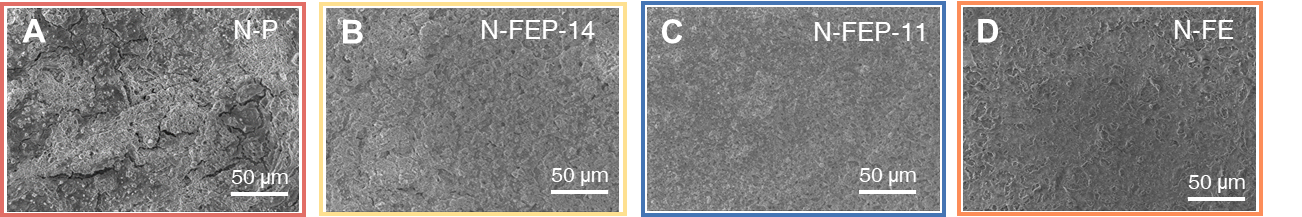


**Figure S14.** SEM images of the cycled Na metal electrode using the (A) N-P, (B) N-FEP-14, (C) N-FEP-11 and (D) N-FE electrolytes. The Na metal was retrieved from Na/Cu cells for after 20 cycles at cycling condition of 0.5 mA/cm^2^, 1.0 mAh/cm^2^.


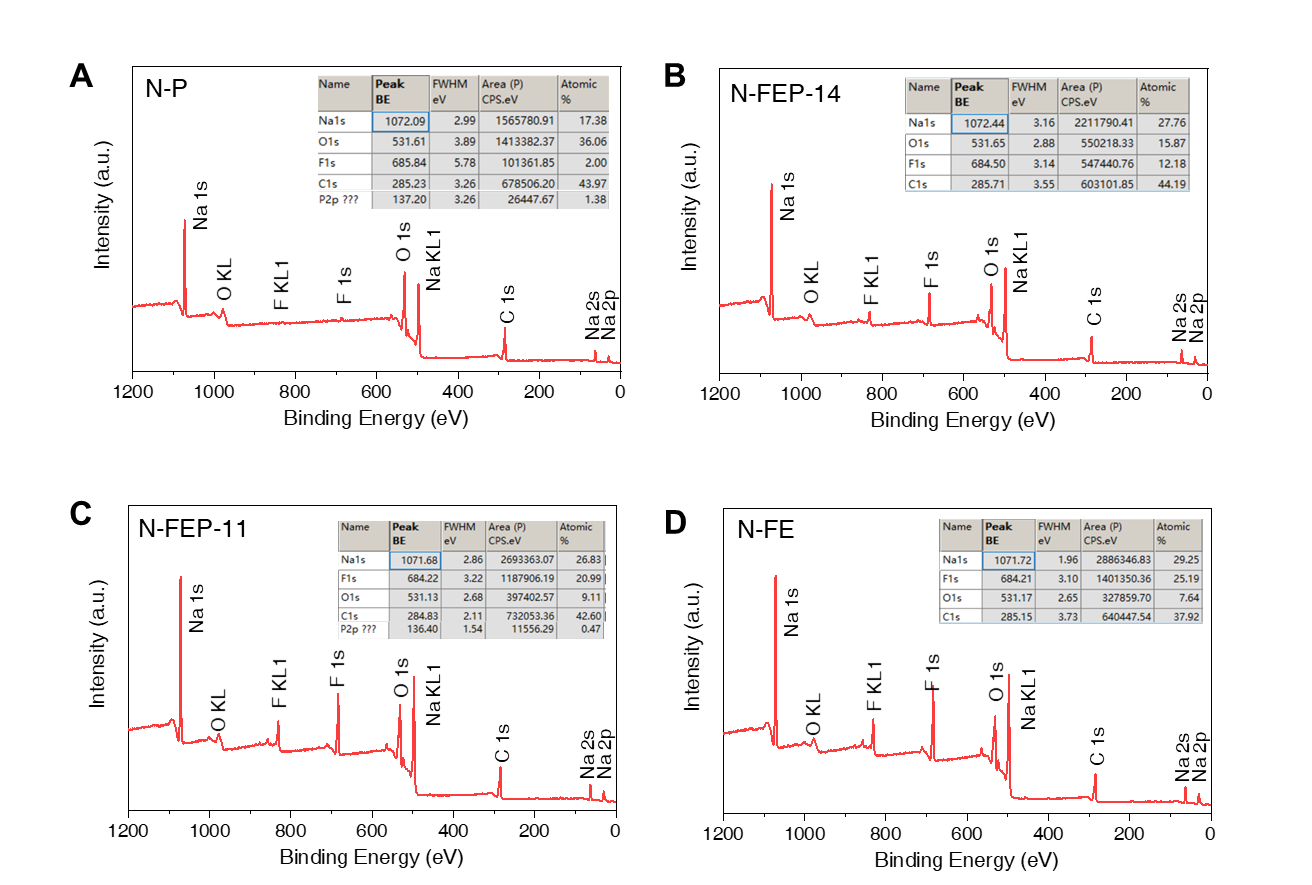


**Figure S15.** Full survey XPS analysis on the surface of the deposited Na using the (A) N-P, (B) N-FEP-14, (C) N-FEP-11 and (D) and N-FE electrolytes, showing the steadily increasing ratio of the F element as the FEC concentration increases.


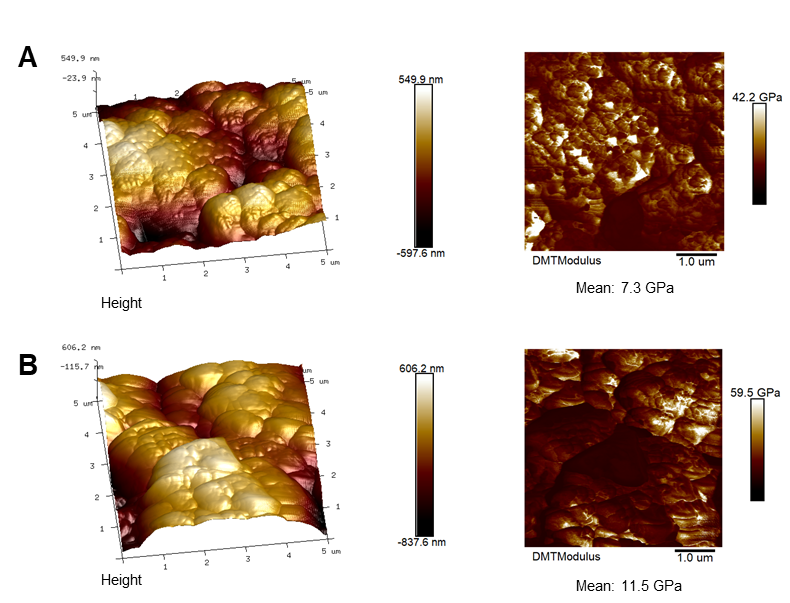


**Figure S16.** AFM characterization on the topography and Young’s modulus of the deposited Na surface using the (A) N-P and the (B) N-FEP-14 electrolytes, with the mean Young’s modulus marked below.


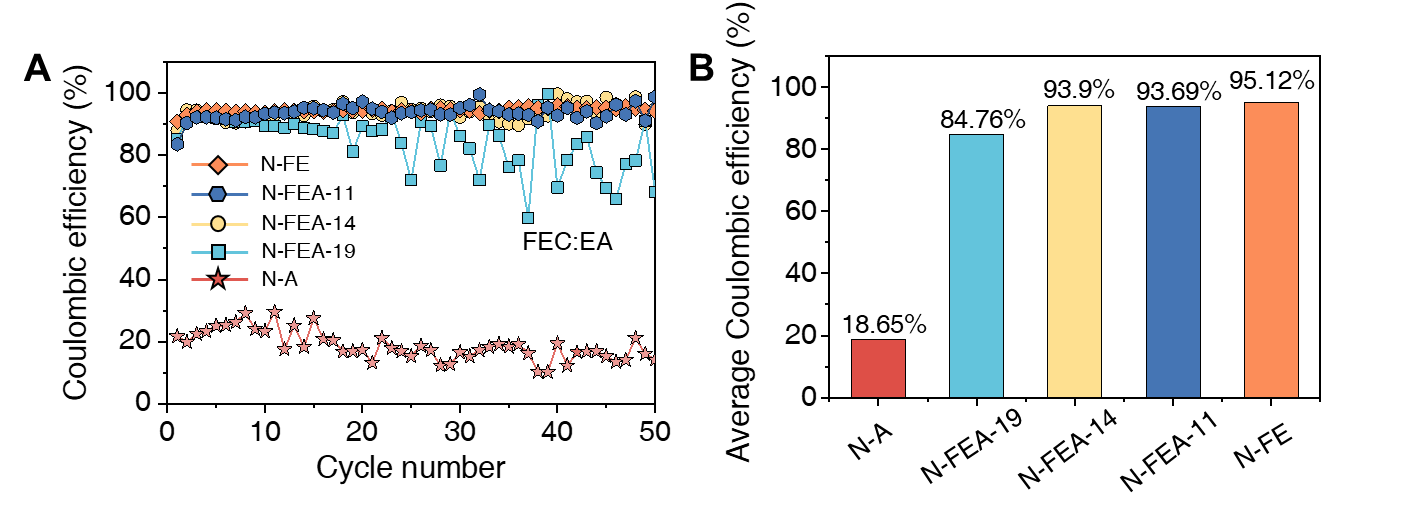


**Figure 17.** (A) The CE for Na plating/stripping assessed in Na/Cu cells using 1.0 M NaPF_6_ in FEC/EA binary solvents (denoted as N-FEA) at the corresponding volumetric ratios. The cycling condition was 0.5 mA/cm^2^, 1.0 mAh/cm^2^. (B) The calculated average Na CE in the corresponding electrolyte.


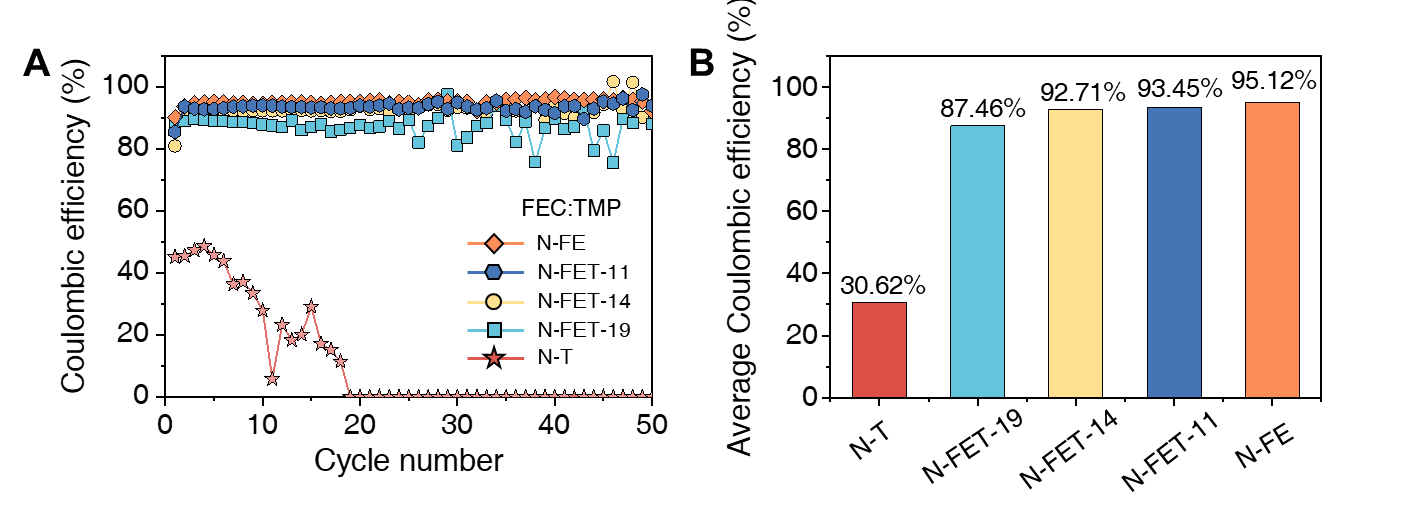


**Figure S18.** (A) The CE for Na plating/stripping assessed in Na/Cu cells using 1.0 M NaPF_6_ in FEC/TMP binary solvents (denoted as N-FET) at the corresponding volumetric ratios. The cycling condition was 0.5 mA/cm^2^, 1.0 mAh/cm^2^. (B) The calculated average Na CE in the corresponding electrolyte.


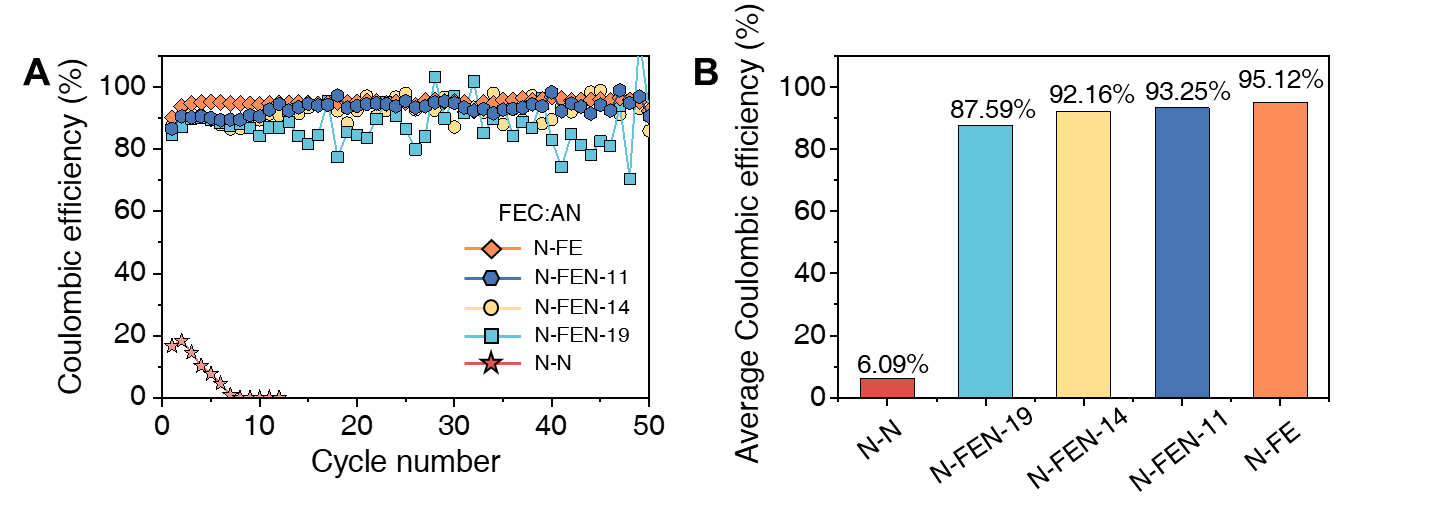


**Figure S19.** (A) The CE for Na plating/stripping assessed in Na/Cu cells using 1.0 M NaPF_6_ in FEC/AN binary solvents (denoted as N-FEN) at the corresponding volumetric ratios. The cycling condition was 0.5 mA/cm^2^, 1.0 mAh/cm^2^. (B) The calculated average Na CE in the corresponding electrolyte.


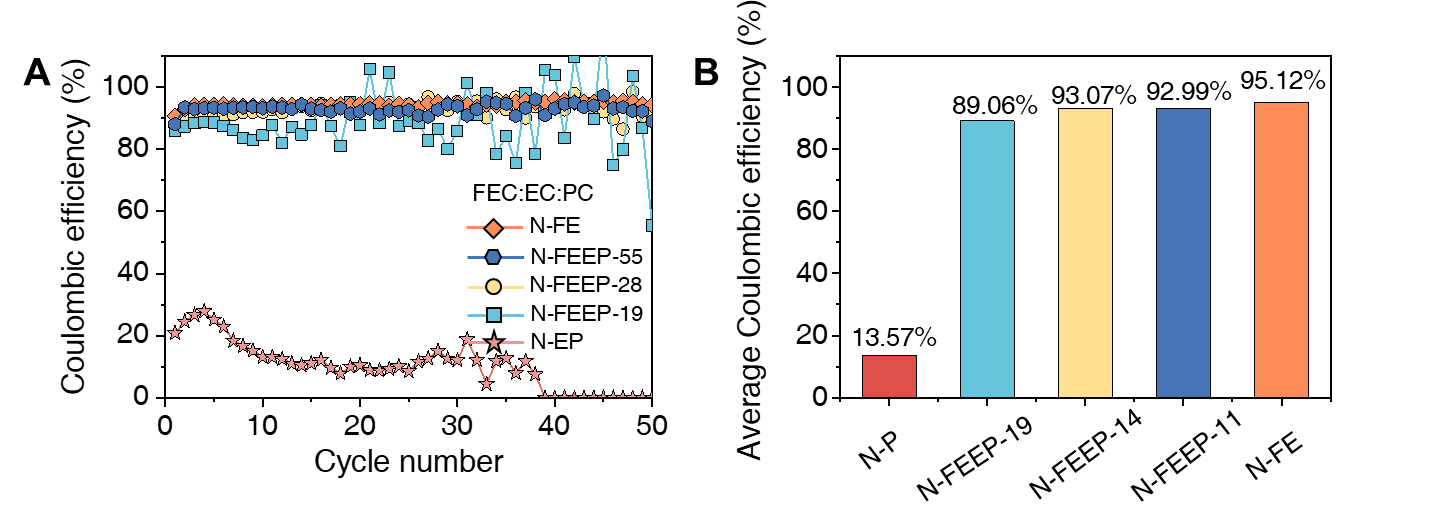


**Figure S20.** (A) The CE for Na plating/stripping assessed in Na/Cu cells using 1.0 M NaPF_6_ in FEC/EC/PC ternary solvents (denoted as N-FEEP) at the corresponding volumetric ratios. The cycling condition was 0.5 mA/cm^2^, 1.0 mAh/cm^2^. (B) The calculated average Na CE in the corresponding electrolyte.

**References**

1. Gu, Z.-Y.; Guo, J.-Z.; Sun, Z.-H.; Zhao, X.-X.; Li, W.-H.; Yang, X.; Liang, H.-J.; Zhao, C.-D.; Wu, X.-L., Carbon-coating-increased working voltage and energy density towards an advanced Na3V2 (PO4) 2F3@ C cathode in sodium-ion batteries. *Sci. Bull.* **2020,** *65* (9), 702-710.

2. Abraham, M.; Murtola, T.; Schulz, R., Pá ll S, Smith JC, Hess B, et al. GROMACS: High performance molecular simulations through multi-level parallelism from laptops to supercomputers. SoftwareX. 2015; 1: 19–25.

3. Sambasivarao, S. V.; Acevedo, O., Development of OPLS-AA force field parameters for 68 unique ionic liquids. *J. Chem. Theory Comput.* **2009,** *5* (4), 1038-1050.

4. Doherty, B.; Zhong, X.; Gathiaka, S.; Li, B.; Acevedo, O., Revisiting OPLS force field parameters for ionic liquid simulations. *J. Chem. Theory Comput.* **2017,** *13* (12), 6131-6145.

5. Dodda, L. S.; Cabeza de Vaca, I.; Tirado-Rives, J.; Jorgensen, W. L., LigParGen web server: an automatic OPLS-AA parameter generator for organic ligands. *Nucleic Acids Res.* **2017,** *45* (W1), W331-W336.

6. Martínez, L.; Andrade, R.; Birgin, E.; Martínez, J., Software news and update packmol: a package for building initial configurations for molecular dynamics simulations. *J. Comput. Chem* **2009,** *30* (13), 2157-2164.

7. Berendsen, H. J.; Postma, J. v.; van Gunsteren, W. F.; DiNola, A.; Haak, J. R., Molecular dynamics with coupling to an external bath. *J. Chem. Phys.* **1984,** *81* (8), 3684-3690.

8. Darden, T.; York, D.; Pedersen, L., Particle mesh Ewald: An N⋅ log (N) method for Ewald sums in large systems. *J. Chem. Phys.* **1993,** *98* (12), 10089-10092.

9. Humphrey, W.; Dalke, A.; Schulten, K., VMD: visual molecular dynamics. *J. Molec. Graphics* **1996,** *14* (1), 33-38.

10. Becke, A. D., Density-functional exchange-energy approximation with correct asymptotic behavior. *Phys. Rev. A* **1988,** *38* (6), 3098.

11. Chengteh Lee, W. Y., and Robert G. Parr, Development of the Colle-Salvetti correlation-energy formula into a functional of the electron density. *Phys. Rev. B* **1988,** *37* (2), 785.

12. Beck, A. D., Density-functional thermochemistry. III. The role of exact exchange. *J. Chem. Phys.* **1993,** *98* (7), 5648-6.

13. Petersson, a.; Bennett, A.; Tensfeldt, T. G.; Al‐Laham, M. A.; Shirley, W. A.; Mantzaris, J., A complete basis set model chemistry. I. The total energies of closed‐shell atoms and hydrides of the first‐row elements. *J. Chem. Phys.* **1988,** *89* (4), 2193-2218.

14. Petersson, G.; Al‐Laham, M. A., A complete basis set model chemistry. II. Open‐shell systems and the total energies of the first‐row atoms. *J. Chem. Phys.* **1991,** *94* (9), 6081-6090.

15. Marenich, A.; Cramer, C.; Truhlar, D., Universal Solvation Model of the Solvent Defined by the Bulk Dielectric Constant and Atomic Surface Tensions. *J. Phys. Chem. B* **2009,** *113*, 6378.

16. Frisch, M.; Trucks, G.; Schlegel, H.; Scuseria, G.; Robb, M.; Cheeseman, J.; Scalmani, G.; Barone, V.; Mennucci, B.; Petersson, G., Gaussian 09, Revision D. 01; Gaussian: Wallingford, CT, 2009.

17. Schäfer, A.; Horn, H., *J. Phys. Chem. Chem. Phys* **2005,** *7*, 3297-3305.
